# Genetic ablation of TRPC6 facilitated age-dependent atherosclerosis progression in an ApoE-/- mouse model

**DOI:** 10.3389/fphys.2026.1884753

**Published:** 2026-07-22

**Authors:** Isaac S. Demaree, Sreepadaarchana Munjuluri, Saketh Boyapalli, Regan E. Beaty, Dru A. Wilkerson, Sruthilatha Sakthi Velavan, Fletcher A. White, Alexander G. Obukhov

**Affiliations:** 1 Department of Anatomy, Cell Biology & Physiology, Indiana University School of Medicine, Indianapolis, IN, United States; 2 Department of Anesthesia, Indiana University School of Medicine, Indianapolis, IN, United States

**Keywords:** atherosclerosis, calcification, cation channels, fibrous cap, TRPC6

## Abstract

**Background:**

TRPC6 channels are expressed in endothelial cells, smooth muscle cells, and macrophages within the atherosclerotic segments of conduit blood vessels. Genetic or pharmacological inactivation of TRPC6 was variably associated with the development of atherosclerosis, a risk factor for myocardial infarction, ischemic stroke, and/or peripheral vascular disease.

**Methods:**

We used two genetically matched mouse strains, ApoE^-/-^;TRPC6^-/-^ and ApoE^-/-^;TRPC6^+/+^, and investigated how genetic ablation of TRPC6 affects age-dependent progression of atherosclerosis in ApoE^-/-^ mice. Aortas were isolated from the mice and subjected to histopathological investigation.

**Results:**

We assessed atherosclerotic lesion progression in mouse aortas by measuring their area, fibrous cap thickness, and calcification. We found that one-year-old mature mice had similar aortic atherosclerosis. However, aged 1.5-year-old ApoE^-/-^;TRPC6^-/-^ mice exhibited significantly greater atherosclerosis than ApoE^-/-^;TRPC6^+/+^ mice. Averaged fibrous cap thickness was also larger in atherosclerotic lesions from ApoE^-/-^;TRPC6^-/-^ mice compared to ApoE^-/-^;TRPC6^+/+^ mice, whereas calcification was not different between the two mouse strains at any age.

**Conclusions:**

Age-dependent, spontaneous atherosclerosis progression was greater in aged but not mature ApoE^-/-^;TRPC6^-/-^ mice compared to ApoE^-/-^;TRPC6^+/+^ mice. Thus, genetic ablation or chronic inhibition of TRPC6 may facilitate the development of spontaneous atherosclerosis in aged ApoE^-/-^ mice.

## 1 Introduction

Atherosclerosis is an inflammatory disease characterized by the formation of atherosclerotic lesions (plaques) in the conduit vasculature, which mainly consist of cholesterol, lipids (fat), macrophages, proliferating smooth muscle cells, and calcium deposits. These lesions can occur as early as childhood, and they advance with age, with several risk factors facilitating atherosclerosis progression (McGill et al., 2000). The risk factors include a high-fat diet, high plasma cholesterol, obesity, physical inactivity, chronic kidney failure, and diabetes mellitus (Moller and Kaufman, 2005). Notably, there is also a strong genetic component underlying the risk of developing atherosclerotic disease (Biros et al., 2008; Stylianou et al., 2012; Zangas and Georgakis, 2026).

Atherosclerotic plaque rupture leads to thrombus formation and may be followed by occlusion of the affected artery, an event resulting in ischemia of the surrounding tissue. In severe cases, thrombotic arterial occlusion may be complete and, if not treated promptly, may result in tissue death, as happens during myocardial infarction and stroke. Atherosclerotic lesion rupture often occurs in vulnerable or high-risk plaques that exhibit a significant necrotic core due to apoptosis and necrosis of macrophages and foam cells, and/or thinning of the fibrous cap (Bentzon et al., 2014). The stability of the fibrous cap depends largely upon its composition and thickness. The thinning of the cap, due to loss of the collagen matrix and smooth muscle cells, may lead to fibrous cap ruptures. Conversely, lesions with thicker fibrous caps are less prone to rupture because strengthening of the fibrous cap helps outweigh the mechanical load of the necrotic core in inflamed atheromas (Wissing et al., 2022). Thus, a thick fibrous cap, with abundant collagen accumulation and migration of proliferating smooth muscle cells at the atheroma-blood interface, is known to stabilize atherosclerotic plaques.

Calcification or calcium content of the atherosclerotic plaque increases during atherosclerosis progression and has important prognostic value (Onnis et al., 2024). For example, the coronary artery calcification (CAC) score is an important clinical characteristic that reflects the risk of adverse cardiovascular events in patients. Extensive calcification is associated with overall atherosclerosis severity; however, the type of calcification, not the amount, has been found to determine plaque vulnerability and the risk of plaque rupture. Atheromas with dense sheet calcification in the core are associated with increased stability, while atheromas with microcalcifications or fragmented calcium are associated with decreased stability or an increased risk for atherosclerotic plaque rupture or erosion (Jinnouchi et al., 2020).

Given the genetic influence on the progression of atherosclerotic disease, there has been interest in studying potentially related genes. One such group is the transient receptor potential canonical (TRPC) family of ion channel subunits. There are seven TRPC proteins, TRPC1 – TRPC7 (Chen et al., 2020). These proteins form cation channels throughout the vasculature and have been implicated in various cardiovascular pathologies (Chen et al., 2020; Saqib et al., 2023). TRPCs are localized to the plasma membrane and are activated in a GPCR/PLC-dependent manner, leading to Na^+^ and Ca^2+^ influx into vascular cells and consequent cell depolarization.

Previous studies have shown that impairment of the TRPC3 channel in mice with ablated apolipoprotein E (ApoE) correlates with an increased risk of atherosclerotic lesion development (Smedlund et al., 2015; Vazquez et al., 2016). TRPC6 is homologous to TRPC3; however, TRPC6 exhibits a different expression pattern in the cardiovascular system compared to TRPC3. The TRPC6 gene is expressed in endothelial cells, vascular smooth muscle cells, and macrophages. Previous studies have shown that deletion of TRPC6 in mice promoted an increase in proinflammatory cytokines as well as smooth muscle cell proliferation, dedifferentiation, and migration, as well as luminal narrowing in the carotid arteries when subjected to an endothelial injury (Oda et al., 2017; Smith et al., 2020). It has been hypothesized that impairment of the TRPC6 channel can lead to certain cardiovascular diseases, such as atherosclerosis and myocardial infarction (Saqib et al., 2023).

TRPC6 and TRPC1 are the two main channels expressed in vascular smooth muscle cells, as well as endothelial cells and macrophages. Mouse models with the ApoE^-/-^ genetic background are typically used to study atherosclerosis progression because these mice exhibit elevated blood plasma total cholesterol even when fed standard chow. As a result, ApoE^-/-^ mice have a greater propensity to develop atherosclerosis spontaneously in an age-dependent manner (Zhang et al., 1992).

In this study, we sought to further explore the effect of TRPC6 deletion on the development of age-dependent atherosclerosis. We crossed TRPC6^-/-^ and ApoE^-/-^ mice to create two strains of mice, ApoE^-/-^;TRPC6^-/-^ and ApoE^-/-^;TRPC6^+/+^, and investigated whether ablation of TRPC6 affects atherosclerosis progression in the aortas of mice fed a standard, non-atherogenic diet. The main focus was to determine the relative amount of atherosclerotic plaque development in the aortic arch of TRPC6^-/-^ mice compared to genetically matched TRPC6^+/+^ mice. We also measured the length and thickness of the fibrous caps in atherosclerotic lesions because thinning of the fibrous cap marks the vulnerability of plaques to rupture.

## 2 Methods

### 2.1 Animals

All animal experiments were performed in accordance with an Indiana University School of Medicine’s IACUC-approved protocol. Mice were euthanized using inhalation of carbon dioxide, followed by decapitation and bilateral pneumothorax. The heart and thoracic aorta were removed and cleaned of connective tissue and fat. Mice were housed at the Indiana University Laboratory Animal Resource Center with a 12-hour light/dark cycle in an air-conditioned room. Mice were fed standard chow (Tekland Global 2018SX diet, containing 18% crude protein, <5% crude fat, and 5% crude fiber based on ground wheat and corn).

### 2.2 Development of double knock-out mice

To generate double knock-out mice lacking functional TRPC6 and ApoE, we crossed B6;129S-Trpc6tm1Lbi/Mmjax mice (MMRRC Strain #: 037345-JAX, The Jackson Laboratory, USA) and 129S6/SvEv-Apoetm4Mae/J mice (Strain #: 014556, developed by Dr. Nobuyo N. Maeda laboratory). The mouse TRPC6^-/-^ strain was developed by Dr. Lutz Birnbaumer’s laboratory. TRPC6^-/-^ mice still express TRPC6, but the channel is non-functional because it lacks the pore region, including the transmembrane segments 4 and 5. The 129S-Maeda-ApoE^-/-^ mice uniquely exhibit spontaneous age-dependent atherosclerosis in the aortic arch rather than at the aortic root as usually observed in the typical strain of ApoE^-/-^ on the C57Bl6 genetic background. Initially, we obtained heterozygous mice ApoE^+/-^;TRPC6^+/-^ by crossing ApoE^-/-^ and TRPC6^-/-^ mice. The heterozygous mice were then again crossed to obtain homozygous mice. Littermate offspring pups were genotyped to identify mice with the knockout traits of ApoE^-/-^;TRPC6^-/-^ and ApoE^-/-^;TRPC6^+/+^. Littermate double knockout ApoE^-/-^;TRPC6^-/-^ and knockout ApoE^-/-^;TRPC6^+/+^ pups were isolated and then bred in-house. In this study, we referred to ApoE^-/-^;TRPC6^+/+^ mice as control TRPC6^+/+^ mice, whereas to ApoE^-/-^;TRPC6^-/-^ mice as TRPC6^-/-^ mice. All mice were euthanized by carbon dioxide inhalation with a flowmeter set to deliver about 40-70% of chamber volume per minute, and body weights were measured. For each strain of mice, we tested two age groups: mature mice (200-425 days old) and aged mice (426-550 days old). When identifying age groups, we used the definitions of mouse age provided by the Jackson Laboratory (JAX mice, USA; https://www.jax.org/news-and-insights/jax-blog/2017/november/when-are-mice-considered-old) and the “mouse age to human age chart” available at https://www.animallama.com/mice/pet-mice-lifespan/. Totally, we assessed 20 TRPC6^+/+^ and 35 TRPC6^-/-^ mice.

### 2.3 Cholesterol measurements

In a subset of tested mice, blood samples were collected before euthanasia by cardiac puncture with a 27 G needle attached to a 1 ml monojet tuberculin syringe. After 15 minutes of incubation at room temperature, serum was isolated by centrifugation at 1500 g. Serum cholesterol levels were measured at the Indiana University School of Medicine Translational Core using a Roche Integra 400 analyzer.

### 2.4 Von Kossa staining, Masson’s trichrome staining, and immunohistochemistry

The aortas from TRPC6^-/-^ and TRPC6^+/+^ mice were isolated, cleaned of fat and connective tissue, and fixed in 10% neutral phosphate-buffered formalin. The isolated and cleaned aortas were placed in cassettes and sent to the IU Pathology Immunohistochemistry core for paraffin embedding, sectioning, and staining. The 5 μm thick longitudinal sections of aortas were stained with α-smooth muscle actin (SMA) antibody, Von Kossa (VK) stain, or Masson’s Trichrome (MTC) stain.

The DAKO Masson’s Trichrome Stain and Von Kossa kits were used on the Artisan™ Link Special Stains Instrument (Dako North America, Inc., Agilent Technologies, Carpinteria, CA, USA) for MTC and VK staining. Collagen was stained blue, whereas SMCs were stained red. The α-smooth muscle actin antibody (dilution 1:50, α-SMA, human, Sigma, St. Louis, MO, USA) was used to stain SMC in the atheromas. The sections were incubated with the primary antibodies at room temperature and then probed using the DAKO Flex system, followed by the DAKO LSAB2 horseradish peroxidase-conjugated polymer. The DAKO wash buffer was used for all washes. Diaminobenzidine was used to develop the brown color.

### 2.5 Section imaging

Sections were examined using a Zeiss inverted microscope. The stained aortic sections were placed on the microscope stage and photographed using a 10x objective with a numerical aperture of 0.25. Individual histology images from each aorta were stitched together using CorelDraw before analysis (**Supplementary Figure 1**). The degree of atherosclerosis and the length of the fibrous cap were then determined using Image-Pro Premier 9.3. Lumen and atheroma areas were measured using the polygon tool of Image-Pro, and atheroma percentage relative to the overall lumen area was calculated. Fibrous cap length was also measured within the atheroma and was normalized by the total linear measurement of the atheroma surface to calculate a percentage. Calcification, collagen, and smooth muscle cell content were determined using the Smart Segmentation tool in Image-Pro and were normalized to atheroma area. For representative histological images, background subtraction was used in Image-Pro.

### 2.6 Statistical analyses

SigmaPlot 12.5 was used for data analysis, comparing TRPC6^+/+^ and TRPC6^-/-^ mouse sections. The two-tailed t-test was used to determine whether there was a significant difference between the tested groups when the data sets were normally distributed. The Mann-Whitney Rank Sum Test was used to determine whether there is a significant difference between data sets that did not have a normal distribution. Data analyses were performed by three independent experimenters, who were not blinded to the genotype. 8 to 14 images from each of the aortic arches were analyzed and averaged to obtain the individual mouse %atheroma value. These values were then averaged. The percent of atheroma area (%atheroma) was determined in SMA-, Von Kossa-, and MTC-stained sections.

## 3 Results

### 3.1 Weights and serum total cholesterol levels of tested mice

**Figure 1:**
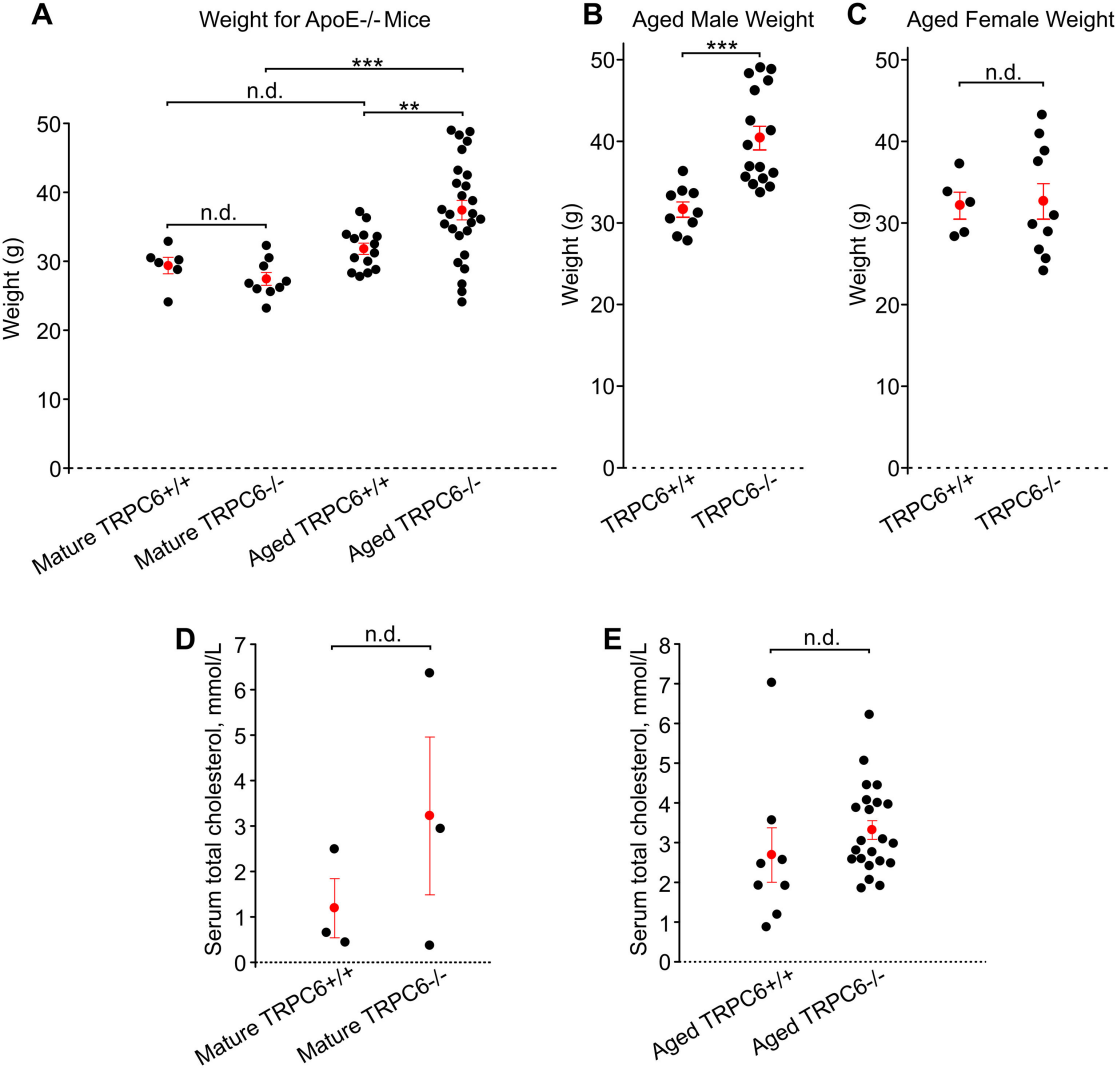
Body weights and serum total cholesterol levels in TRPC6-/- and TRPC6+/+ mice. **(A)** Body weight comparison of mature and aged TRPC6^+/+^ (n=6 and n=14, respectively) and TRPC6^-/-^ (n=9 and n=26, respectively) mice. **(B, C)** Sex-stratified comparison of body weights in both genotypes in aged male (B, n=9 for TRPC6^+/+^ and n=16 for TRPC6^-/-^) and female (C, n=5 for TRPC6^+/+^ and n=10 for TRPC6^-/-)^ mice. **(D, E)** Serum total cholesterol levels in mature and aged TRPC6^+/+^ (n=3 and n=8, respectively) and TRPC6^-/-^ (n=3 and n=22, respectively) mice. Two-tailed t-tests were used for normal data sets, and the Mann-Whitney Rank Sum Test for non-normal data sets. Black-filled circles show individual weight measurements, and the red-filled circle corresponds to the mean value ± SEM. **p < 0.01. ***p < 0.001. n.d. = no significant difference.

Weight was measured before each mouse was sacrificed and recorded for analysis. Each age group was analyzed separately. When comparing mature mice, there was no significant difference in weight between mature TRPC6^+/+^ and TRPC6^-/-^ mice (29.4 ± 1.2 g versus 27.4 ± 0.9 g, **Figure 1A**). However, in the aged group, TRPC6^-/-^ mice weighed significantly more than TRPC6^+/+^ mice (37.4 ± 1.4 g vs. 31.8 ± 0.8 g; p = 0.007, **Figure 1A**). Comparing mature versus aged TRPC6^-/-^ mice, aged mice were also heavier (27.4 ± 0.9 g vs. 37.4 ± 1.4 g; p < 0.001, **Figure 1A**). This difference was not observed when comparing the two age groups in TRPC6^+/+^ mice.

Noting the increased weights in TRPC6^-/-^ mice, we then stratified the data by sex. The difference between TRPC6^+/+^ and TRPC6^-/-^ male mice was still present in the aged male mice (31.7 ± 0.9 g vs. 40.4 ± 1.4 g; p=0.00031, **Figure 1B**). Interestingly, the difference in weight between TRPC6^+/+^ and TRPC6^-/-^ genotypes was not observed in aged female mice (32.1 ± 1.6 g vs 32.6 ± 2.2 g; p=0.878, **Figure 1C**).

Additionally, total cholesterol levels were determined in a subset of mice. However, no significant difference was found between serum total cholesterol levels in the tested groups of mature (1.2 ± 0.7 mmol/L, 3.2 ± 1.7 mmol/L, TRPC6^+/+^ versus TRPC6^-/-^, respectively, p = 0.335; **Figure 1D**) or aged (2.7 ± 0.7 mmol/L, 3.3 ± 0.2 mmol/L, TRPC6^+/+^ versus TRPC6^-/-^, respectively, p = 0.071; **Figure 1E**) mice.

### 3.2 Atheroma burden

**Figure 2:**
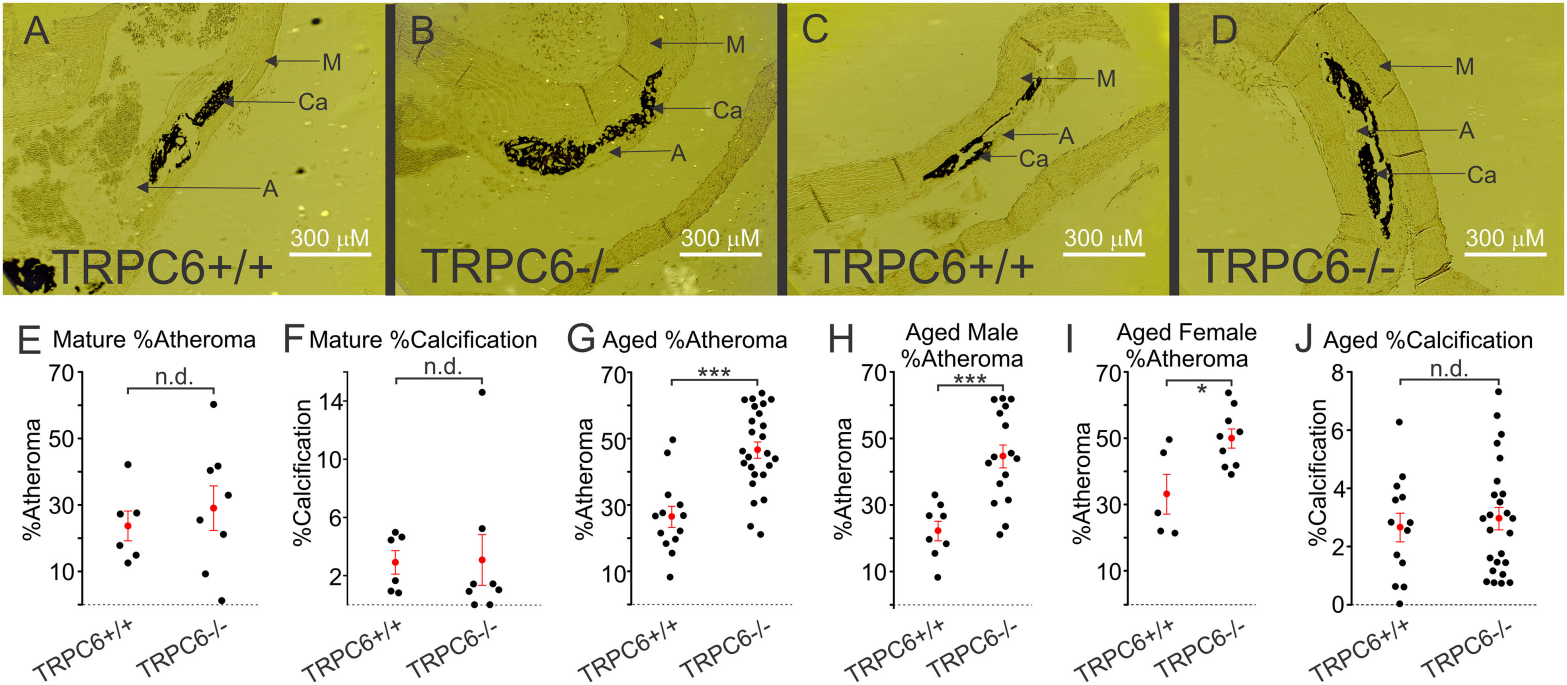
TRPC6 deletion increases atheroma burden without affecting calcification in an age-dependent manner. **(A–D)**. Representative Von Kossa-stained aortic sections from mature mice with both TRPC6^+/+^ **(A)** and TRPC6^-/-^ **(B)** genotypes and aged mice with both TRPC6^+/+^ **(C)** and TRPC6^-/-^ **(D)** genotypes. **(E, F)** Quantification of atheroma burden and calcification in aortic sections from mature mice shows no significant differences between TRPC6^-/-^ and TRPC6^+/+^ groups (in **(E)**, TRPC6^+/+^: n=6; TRPC6^-/-^: n=8; in **(F)**, TRPC6^+/+^: n=6; TRPC6^-/-^: n=8, respectively). **(G)** In aged mice, TRPC6^-/-^ mice exhibit increased atheroma burden compared to controls (TRPC6^+/+^: n=13; TRPC6^-/-^: n=25), even among sex-stratified male **(H),** TRPC6^+/+^: n=8; TRPC6^-/-^: n=16) and female **(I),** TRPC6^+/+^: n=5; TRPC6^-/-^: n=9) groups. **(J)** The analysis of Von Kossa-stained aortic sections from aged mice shows that calcification remains unchanged (TRPC6^+/+^: n=13; TRPC6^-/-^: n=25). Two-tailed t-tests were used for normal data sets, and the Mann-Whitney Rank Sum Test for non-normal data. Data are presented as mean ± SEM (red-filled circle and red error bars) with individual data points shown as black-filled circles. M, tunica media; Ca, calcification; A, atheroma. ***p < 0.001. n.d. = no significant difference.

**Figure 3:**
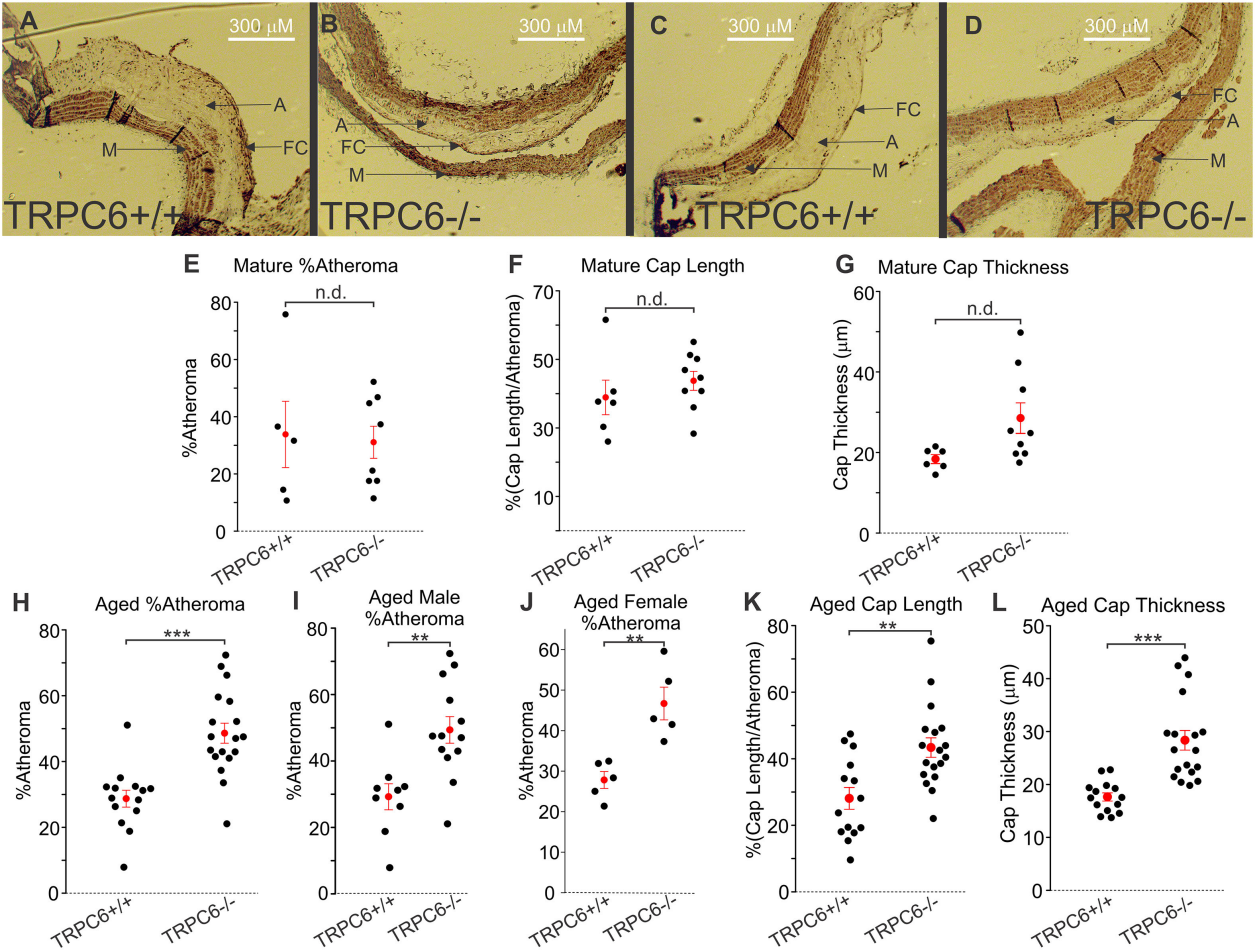
TRPC6 deletion increases fibrous cap formation in an age-dependent manner (SMA staining). **(A–D)** Representative SMA-stained aortic sections from TRPC6^-/-^ and TRPC6^+/+^ mice in mature **(A, B)** and aged groups (**C, D, E-L).** Quantification of atheroma burden **(E, H)**, sex-stratified %atheroma. **(I, J)**, fibrous cap length **(K)**, and fibrous cap thickness **(L)** in aortic sections from mature **(E-G)** and aged **(H-L)** in TRPC6^-/-^ and TRPC6^+/+^ groups (in **(E)**, TRPC6^+/+^: n=5 and TRPC6^-/-^: n=8; in **(F)**, TRPC6^+/+^: n=6 and TRPC6^-/-^: n=9; in **(G)**, TRPC6^+/+^: n=6 and TRPC6^-/-^: n=9; in **(H)**, TRPC6^+/+^: n=14 and TRCP6^-/-^: n=18; in **(I)**, TRPC6^+/+^: n=9 and TRPC6^-/-^: n=13; in **(J)**, TRPC6+/+: n=5 and TRPC6^-/-^: n=5; in **(K)**, TRPC6 ^+/+^: n=14 and TRPC6^-/-^: n=18; in **(L)**, TRPC6^+/+^; n=14 and TRPC6^-/-^: n=18). Two-tailed t-tests were used for normally distributed data, and the Mann-Whitney Rank Sum Test for non-normally distributed data. M, tunica media; FC, fibrous cap; A, atheroma. Data are presented as mean ± SEM (red-filled circle and red error bars) with individual data points shown as black-filled circles. **p < 0.01. ***p < 0.001. n.d. = no significant difference.

**Figure 4:**
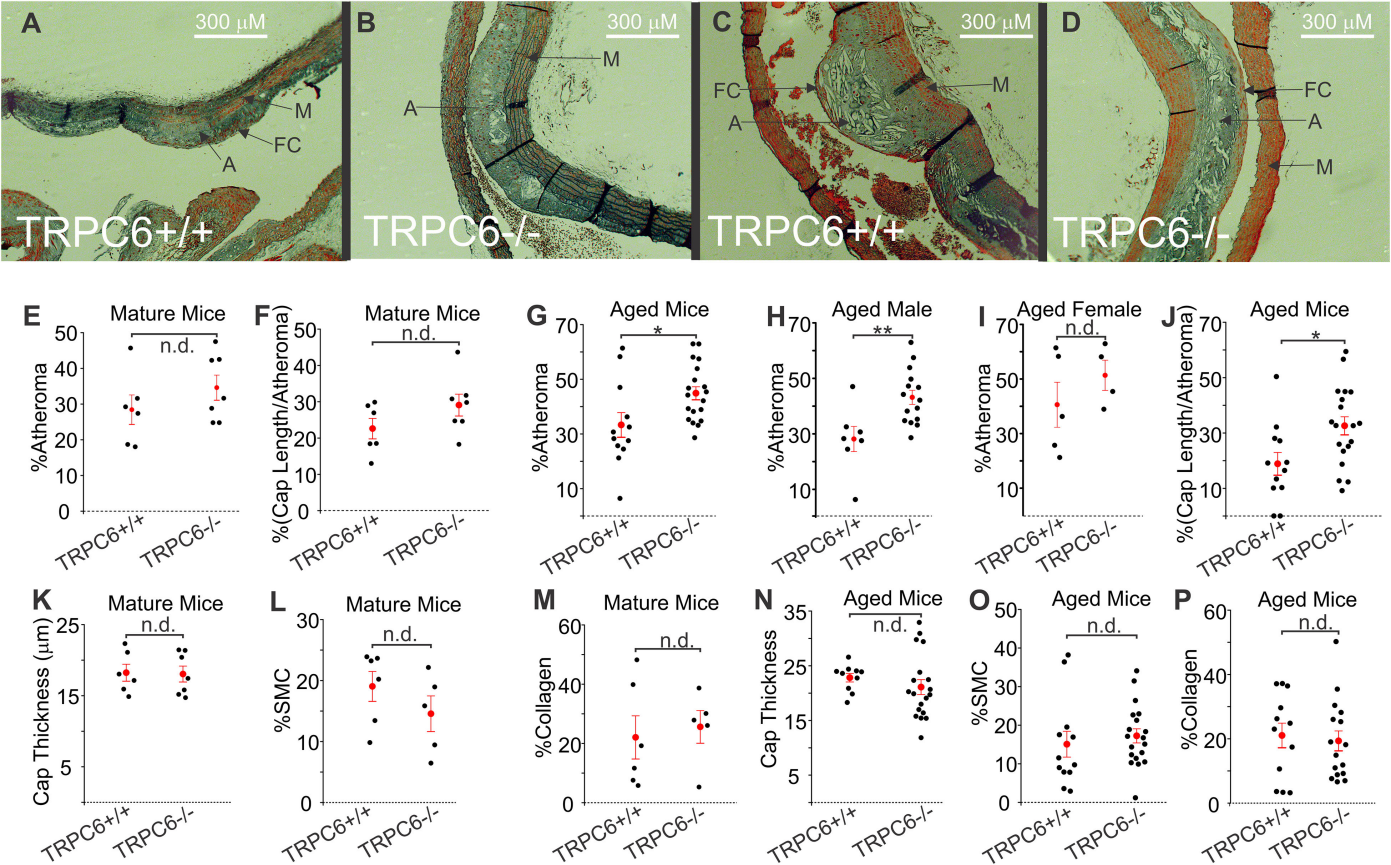
TRPC6 deletion alters fibrous cap formation without affecting collagen or smooth muscle cell content (MTC staining). **(A–D)** Representative Masson’s Trichrome (MTC)-stained aortic sections from mature and aged TRPC6^+/+^ and TRPC6^-/-^ mice. (**E, F, K–M**) Quantification of atheroma burden **(E),** TRPC6^+/+^: n= 6; TRPC6^-/-^: n= 7), fibrous cap length (% of atheroma length, (**F),** TRCP6^+/+^: n=6; TRPC6^-/-^: n=7), fibrous cap thickness **(K),** TRPC6^+/+^: n= 6; TRPC6^-/-^: n= 7), smooth muscle cell (SMC, **(L),** TRPC6^+/+^: n= 6; TRPC6^-/-^: n= 5) content, and collagen content **(M),** TRPC6^+/+^: n= 6; TRPC6^-/-^: n= 5) in aortic sections from mature mice in both TRPC6^-/-^ and TRPC6^+/+^ groups. (**G–J).** Quantification of atheroma burden **(G),** TRPC6^+/+^: n= 12; TRPC6^-/-^: n= 19), sex-stratified %atheroma (in **(H)**, TRPC6^+/+^: n= 7 and TRPC6^-/-^: n= 15; in **(I),** TRPC6^+/+^: n= 5; TRPC6^-/-^: n= 4), fibrous cap length (% of atheroma length, **(J),** TRPC6^+/+^: n= 12; TRPC6^-/-^: n= 19, fibrous cap thickness **(N),** TRPC6^+/+^: n= 10; TRPC6^-/-^: n= 19), smooth muscle cell (SMC) content **(O),** TRPC6^+/+^: n= 12; TRPC6^-/-^: n= 19), and collagen content **(P),** TRPC6^+/+^: n= 12; TRPC6^-/-^: n= 16) in aortic sections from aged mice in both genotypes. Two-tailed t-tests were used for normally distributed data sets, and the Mann-Whitney Rank Sum Test for non-normally distributed data. M, tunica media; FC, fibrous cap; A, atheroma. Data are presented as mean ± SEM (red-filled circle and red error bars) with individual data points shown as black-filled circles. *p < 0.05, **p < 0.01. n.d. = no significant difference.

To assess the progression of atherosclerotic lesions in the tested mice, the aortic arches were stained in triplicate with the aforementioned immunohistochemical stains. It was found that among the mature cohorts, there was no significant difference in atheroma burden between TRPC6^-/-^ and TRPC6^+/+^ for any of the analyzed genotypes (atheroma/lumen % in SMA-stained sections: 29.5 ± 5.2% vs 35.9 ± 9.7%, p=0.536). However, we found that TRPC6^-/-^ mice showed greater atheroma development compared with TRPC6^+/+^ mice (atheroma/lumen % for VK stained sections: 46.5 ± 2.5% vs 26.4 ± 3.2%; p=0.00002; atheroma/lumen % for SMA stained sections: 48.6 ± 3.0% vs 28.7 ± 2.6%, p= 0.00004; atheroma/lumen % for MTC stained sections: 44.9 ± 2.4 vs 33.3 ± 4.5, p=0.0187) among the aged group for both sexes (**Figures 2**–**4**). We did not find a difference between mature and aged TRPC6^+/+^ mice for %atheroma (23.7 ± 4.5% vs 26.4 ± 3.2%; p=0.631).

### 3.3 Atheroma calcification

The calcification of the aorta atheromas was assessed using the Von Kossa stain, which reveals calcium deposits in the vascular wall. The areas of calcification are darkly stained and easily identified (**Figure 2**). We measured the stained areas as a percentage of the overall atheroma area and found no significant differences between the TRPC6^-/-^ and TRPC6^+/+^ genotypes in either the mature (2.8 ± 1.6% vs 2.9 ± 0.8%, p=0.376) or aged (2.9 ± 0.4% vs 2.6 ± 0.5%, p=0.636) mouse groups. There was also no difference in %calcification between mature and aged TRPC6^+/+^ mice (2.9 ± 0.8% vs 2.6 ± 0.5%, p=0.091).

### 3.4 Fibrous cap

The fibrous caps of the atheroma were visualized with both SMA and MTC stains. In sections of the aortas of mature mice stained with SMA (**Figure 3**), no significant difference was found between fibrous cap length, presented as a percentage relative to the length of the atheroma (43.8 ± 2.8% vs 38.9 ± 5.0%; p=0.377), or average thickness of the cap (28.5 ± 3.8 µm vs 18.4 ± 1.1 µm; p=0.053) in mature TRPC6^-/-^ and TRPC6^+/+^ mice. In aged mice (**Figure 3**), a significant difference was seen between TRPC6^-/-^ and TRPC6^+/+^ mice in both relative fibrous cap length (43.4 ± 2.9% vs 28.1 ± 3.3%; p = 0.002) and average thickness of the fibrous cap (28.3 ± 1.9 µm vs 17.6 ± 0.8 µm; p < 0.001) in both male and female mice. No difference was found between average fibrous cap length (38.9 ± 5.0% vs 28.1 ± 3.3%; p=0.089) and average thickness of the fibrous cap (18.4 ± 1.1 µm vs 17.6 ± 0.8 µm, p=0.091) in mature and aged TRPC6^+/+^ mice.

For the MTC stain, there was again no difference in the mature age group between TRPC6^-/-^ and TRPC6^+/+^ mice (Relative fibrous cap length: 29.1 ± 3.0% vs 22.6 ± 2.8%, p = 0.148; Fibrous cap thickness: 18.0 ± 1.1 µm vs 18.2 ± 1.2 µm, p=0.913; **Figure 4**). In the aged group (**Figure 4**), there was again a difference in relative fibrous cap length (32.6 ± 3.3% vs 18.9 ± 4.1%; p = 0.014, **Figure 4**), but no difference was seen in the average thickness between the TRPC6^-/-^ and control TRPC6^+/+^ mice (21.1 ± 1.4 µm vs 22.8 ± 0.8 µm, p=0.103).

### 3.5 Collagen and SMC composition

Using the smart segmentation tool in Image-Pro, we estimated the collagen and smooth muscle cell (SMC) content within the aorta atheromas. We did not see any changes in either collagen or SMC composition in the atheromas (**Figure 4**). There was no significant difference between the TRPC6^-/-^ and TRPC6^+/+^ in mature (SMC: 14.6 ± 2.9 vs 19.0 ± 2.4, p=0.531; Collagen: 25.6 ± 5.5 vs 22.0 ± 7.3, p=0.716) or aged mice (SMC: 17.2 ± 1.8 vs 15.1 ± 3.4, p=0.554; Collagen: 19.3 ± 3.1 vs 21.0 ± 3.8, p=0.731).

## 4 Discussion

In this study, we compared spontaneous atheromas in mature and aged TRPC6^-/-^ and TRPC6^+/+^ mice, both of which had the ApoE^-/-^ genetic background. The weight of the TRPC6^-/-^ mice was greater than that of the TRPC6^+/+^ control mice in the aged groups. Furthermore, there was a sex difference, with only TRPC6^-/-^ male mice being heavier than control mice. No weight difference was observed in female mice or in the mature group for both sexes. These data suggest that the ablation of the TRPC6 protein may lead to increased age- and sex-dependent weight gain. This could indicate a broader metabolic effect of TRPC6 in the aged body. Interestingly, an increased BMI and adiposity are directly correlated with an increased incidence of cardiovascular diseases (Lemieux and Despres, 2023). It is also possible that weight gain may be one of the mechanisms by which TRPC6 affects atheroma development, a possibility we further analyzed in our histological studies. The sex-stratified analysis suggests that this change in weight is more significant in aged male mice, which could mean a sex-specific role for TRPC6 in weight homeostasis. This age-dependent development is particularly important given that the aged population is rapidly increasing in the US, and the aged people have a higher prevalence of geriatric diseases related to atherosclerosis. Notably, there was no significant difference between serum total cholesterol levels in TRPC6^+/+^ and TRPC6^-/-^ mice from any of the tested groups.

It has already been suggested that TRPC channels, specifically TRPC1, TRPC3, TRPC4, TRPC5, and TRPC6, may be involved in atherosclerosis development, but the relationship is not fully understood (Chen et al., 2020; Saqib et al., 2023). There is also the consideration of whether atherosclerotic changes appear to be age-related, diet-related, or both. Both are factors in the development of atherosclerotic disease, but there is evidence that atherosclerosis is related to extracellular matrix (ECM) changes that happen with aging, and these changes can happen independently of diet (Kohn et al., 2015). Furthermore, it has been suggested that there are unique ECM changes in subjects with age-related atheroma compared to those on atherogenic diets, suggesting that there are differences in the mechanisms of age- and diet-dependent atherosclerosis (Watson et al., 2021).

There is no consensus in the current literature on whether TRPC6 is pro- or anti-atherogenic. Several reports suggested that increased TRPC6 expression is seen in vascular disease states and could be a contributor to atherosclerosis development, perhaps through impaired healing (Bergdahl et al., 2005; Thilo et al., 2012; Rosenbaum et al., 2015). There are also studies that suggest TRPC6 could be a potential target for endothelial pathologies (Zhang et al., 2015; Li et al., 2017; Numaga-Tomita et al., 2019). Oppositely, there is also evidence that TRPC6 could, in fact, play a protective role in the development of atherosclerosis, including in traumatic brain injury-associated endothelial dysfunction and protection from the SMC dedifferentiation that is central to atherosclerosis development (Chen et al., 2019; Smith et al., 2020).

Given the existing disagreement in the literature, this project was designed to directly measure the histological findings of age-related atherosclerosis in TRPC6^-/-^ mice. We found evidence supporting the claim that TRPC6 expression is protective against atheroma development, at least in aged animals, and that, therefore, deletion of this protein leads to increased atherosclerosis in an ApoE^-/-^ mouse model. This could perhaps be explained by the fact that increased TRPC6 expression is an adaptive response to vascular injury and helps mitigate maladaptive changes. Thus, TRPC6 knockout would remove that response to injury, leading to worsening atherosclerosis. All mice in our study were fed standard chow, and age-dependent atherosclerosis was spontaneous. Possibly, TRPC6 could specifically interfere with age-dependent pro-atherosclerotic changes and may play a different role in diet-mediated disease.

As mentioned herein, calcification burden is a clinical measurement that can help prognosticate atherosclerotic changes in living patients. However, at the microscopic level, the specific morphology of atheroma calcifications appears to play a role in the stability of these lesions. This is crucial because a stable atheroma may not cause symptoms until 50% or more of the vessel is occluded (Tamarappoo et al., 2010). On the other hand, an unstable plaque can rupture, occlude downstream vessels following arterial thrombosis, and lead to devastating outcomes such as cerebrovascular accident (CVA) or myocardial infarction (MI). Studies suggest that microcalcifications correlate with instability, whereas larger macrocalcifications are found in more stable lesions (Shioi and Ikari, 2018; Jinnouchi et al., 2020; Shi et al., 2020). Our study examined calcification and found no difference between TRPC6^-/-^ and control TRPC6^+/+^ mice, suggesting that TRPC6 may not play a significant role in overall calcification of atherosclerotic lesions.

Another important factor in determining plaque stability is the fibrous cap. This is a layer of SMCs that organizes on the outside of an atheroma and provides stability to the lesion. Studies have explored this and found that thicker fibrous caps lead to more stable atheromas that are less prone to rupture and to downstream CVA or MI events (Lee, 2000; Virmani et al., 2003; Finet et al., 2004; Liu et al., 2022). Our SMA-staining results showed that in the aged mice, the fibrous cap was thicker and longer in TRPC6^-/-^ mice, suggesting that TRPC6 knockout mice might have larger but more stable atheromas. However, the MTC analysis showed no significant difference in cap thickness, in contrast to the SMA results. This could be explained, in part, by differences in processing between SMA and MTC stains. Nevertheless, we cannot rule out that the vulnerability of atherosclerotic plaques is unaffected by TRPC6 deletion.

Collagen and SMCs are important components in an atheroma and are thought to contribute to the stability of atherosclerotic lesions. It is thought that collagen provides structural support to the atheromas and leads to stable lesions that are less likely to rupture (Adiguzel et al., 2009; Hansson et al., 2015). The same is thought for SMCs, which fits logically since the fibrous cap is composed mostly of these cells. While SMCs are mainly thought to have a positive impact on plaque stability, there is some evidence that suggests that whether they are beneficial or harmful can be dependent on the specific cellular environment (Harman and Jorgensen, 2019). Our analysis of collagen and SMC content in relation to TRPC6 function revealed no significant differences. This suggests that, while TRPC6 deletion affects atheroma development, it does not alter collagen or SMC content in atherosclerotic lesions.

This study has several limitations. The study was conducted during the COVID-19 lockdown, and some blood draws were not performed, while only formalin-fixed aortas were preserved. Therefore, the mature mouse groups contained fewer blood samples. Therefore, in **Figure 1D**, the small sample size may limit the statistical power of the comparison; accordingly, the comparison results should be interpreted with caution. Additionally, since we used an ApoE^-/-^ mouse strain that exhibits diffuse large atherosclerotic plaques in the aortic arches, we sectioned the paraffin-embedded aortas longitudinally. This precluded us from obtaining cross-sections of the aorta. Furthermore, since our goal was to assess plaque vulnerability by comparing fibrous cap thickness and calcification between the animal groups, we did not use Oil Red O staining, which interferes with subsequent assessments of calcification, smooth muscle cell content, and collagen content. Since we report only histological findings, future work will be needed to conduct live-animal functional and mechanistic studies, which will be described in a separate manuscript. Additionally, metabolic confounders, observed sex-specific effects, and a more detailed analysis of plaque composition will need to be further explored in our future experiments.

Thus, our results suggest that TRPC6 may be involved in slowing the spontaneous, age-dependent development and maturation of atherosclerotic lesions, as TRPC6 knockout increased the overall atheroma burden. However, the change was associated with the stabilizing fibrous cap, increasing lesion stability. There is some uncertainty about how the TRPC6 channel modulates atherosclerosis progression. Therefore, further studies will be needed to fully elucidate the impact and mechanism of this relationship. TRPC6 has been identified as a contributor to kidney disease (Staruschenko et al., 2023), such as focal segmental glomerulosclerosis, and is a potential target for pharmacological intervention (Trachtman et al., 2026). Our data suggest that pharmacological targeting of TRPC6 should be used cautiously in the elderly population, as TRPC6 inhibition may be associated with accelerated spontaneous, age-dependent atherosclerosis.

## Data Availability

The raw data supporting the conclusions of this article will be made available by the authors, without undue reservation.

## Supplementary material

Supplementary Figure 1 Sample stitched images of TRPC6^+/+^ (TRPC6 WT) and TRPC6^-/-^ (TRPC6 KO) aortic arches stained with smooth muscle α-actin antibody. The scale bar is provided for each image.
